# Low-Dose Acetylsalicylic Acid and Mitochondria-Targeted Antioxidant Mitoquinone Attenuate Non-Alcoholic Steatohepatitis in Mice

**DOI:** 10.3390/antiox12040971

**Published:** 2023-04-21

**Authors:** Saadet Turkseven, Cristian Turato, Gianmarco Villano, Mariagrazia Ruvoletto, Maria Guido, Massimo Bolognesi, Patrizia Pontisso, Marco Di Pascoli

**Affiliations:** 1 Unit of Internal Medicine and Hepatology (UIMH), Department of Medicine—DIMED, University of Padova, 35100 Padova, Italy; 2 Department of Pharmacology, Faculty of Pharmacy, Ege University, Izmir 35040, Turkey; 3 Department of Molecular Medicine, University of Pavia, 27100 Pavia, Italy; 4 Department of Surgical, Oncological and Gastroenterological Sciences—DISCOG, University of Padova, 35128 Padova, Italy; 5 Pathology ULSS2, Department of Medicine—DIMED, University of Padova, 31100 Treviso, Italy

**Keywords:** liver injury, steatohepatitis, oxidative stress, mitochondria dysfunction

## Abstract

Non-alcoholic fatty liver disease (NAFLD) is the most common chronic liver disease. NAFLD can evolve from simple fatty liver to non-alcoholic steatohepatitis (NASH), and ultimately, to cirrhosis. Inflammation and oxidative stress, promoted by mitochondrial dysfunction, play a crucial role in the onset and development of NASH. To date, no therapy has been approved for NAFLD and NASH. The aim of this study is to evaluate if the anti-inflammatory activity of acetylsalicylic acid (ASA) and the mitochondria-targeted antioxidant effect of mitoquinone could hinder the progression of non-alcoholic steatohepatitis. In mice, fatty liver was induced through the administration of a deficient in methionine and choline and rich in fat diet. Two experimental groups were treated orally with ASA or mitoquinone. Histopathologic evaluation of steatosis and inflammation was performed; the hepatic expression of genes associated with inflammation, oxidative stress, and fibrosis was evaluated; the protein expression of IL-10, cyclooxygenase 2, superoxide dismutase 1, and glutathione peroxidase 1 in the liver was analyzed; a quantitative analysis of 15-epi-lipoxin A4 in liver homogenates was performed. Mitoquinone and ASA significantly reduced liver steatosis and inflammation by decreasing the expression of TNFα, IL-6, Serpinb3, and cyclooxygenase 1 and 2 and restoring the anti-inflammatory IL-10. Treatment with mitoquinone and ASA increased the gene and protein expression of antioxidants, i.e., catalase, superoxide dismutase 1, and glutathione peroxidase 1, and decreased the expression of profibrogenic genes. ASA normalized the levels of 15-epi-Lipoxin A4. In mice fed with a deficient in methionine and choline and rich in fat diet, mitoquinone and ASA reduce steatosis and necroinflammation and may represent two effective novel strategies for the treatment of non-alcoholic steatohepatitis.

## 1. Introduction

Non-alcoholic fatty liver disease (NAFLD) is the most common chronic liver disease in the Western world. The major risk factors for NAFLD include overweight or obesity, type 2 diabetes mellitus, hypertension, and dyslipidemia; its incidence is increasing due the consistent increase in obesity over the recent years. NAFLD is the result of the accumulation of triglycerides and free fatty acids in hepatocytes as a consequence of insulin resistance, enhanced dietary influx, and increased hepatic lipogenesis. Semiquantitative methods for the histological assessment of steatosis are based on routine stains; the most reproducible method follows the acinar architecture and refers to the percentage of liver parenchyma occupied by steatotic hepatocytes. Histopathological findings range from simple fatty liver to non-alcoholic steatohepatitis (NASH), to fibrosis and, ultimately, cirrhosis. The histological criteria for the diagnosis of NASH include steatosis, hepatocellular injury (usually in the form of ballooning), and lobular inflammation. Lobular inflammation typically occurs with a zone 3 predominance and consists of small foci of inflammatory cell infiltrates composed of lymphocytes, eosinophils, and occasionally polymorphonuclear leukocytes; in addition, Kupffer cell aggregates (microgranulomas) and lipogranulomas may be seen in the lobules. As in other forms of chronic liver disease, fibrosis is not required for the diagnosis of steatohepatitis [1,2]. Although scientific investigations into the pathogenesis of NAFLD/NASH have increased exponentially in recent years, the underlying molecular mechanisms are not fully understood. The increase in saturated and mono-unsaturated fat in patients with NAFLD may cause an inflammatory status through the activation of endogenous signal pathways, resulting in the infiltration of inflammatory cells in the liver and cytokine production [3]. At the moment, despite growing interest in the topic and the numerous ongoing clinical trials, no specific therapies for NAFLD have been approved.

Emerging evidence indicates that hepatic mitochondrial dysfunction also plays a critical role in the pathogenesis of NAFLD. Hepatocytes are rich in mitochondria, which, in addition to their role in the oxidation of glucose and fat for the generation of energy for the cells, have a fundamental role in the generation of reactive oxygen species (ROS). An excessive production of ROS, exceeding the cellular antioxidant capacity, can damage components of the cell such as lipids, proteins, and nucleic acids. This leads to oxidative stress, cytokine release, and, ultimately, cell death, causing inflammation and fibrogenesis. This can be observed in conditions of increased oxidation of free fatty acids such as in NASH [4]. A study has investigated the effects of heterozygosity for the mitochondrial trifunctional protein defect in mice on hepatic oxidative stress, insulin resistance, and the development of NAFLD. Aging mice with the deficiency had higher antioxidant activity, suggestive of increased hepatic oxidative stress, and developed hepatic steatosis with elevated alanine aminotransferase, basal hyperinsulinemia, increased insulin after glucose tolerance test, and reduced tolerance to insulin [5]. In hyperphagic obese rats, reduced hepatic fatty acid oxidation and mitochondrial enzyme activity preceded NAFLD development and insulin resistance, confirming that progressive mitochondrial dysfunction contributes to the natural history of obesity-associated NAFLD [6]. Since lipid accumulation in hepatocytes causes mitochondrial damage, with ROS production that promotes NAFLD progression, mitochondria-targeted antioxidants, by preventing mitochondrial dysfunction, could be another effective strategy for the treatment of NASH [7]. Mitoquinone (MitoQ) is a compound with antioxidant properties that passes through all biological membranes and accumulates within mitochondria [8].

Lipoxins are a metabolite of the arachidonic acid pathway which play an important role in inflammation, producing several anti-inflammatory molecules. Lipoxins can be synthesized by two major routes from arachidonic acid; additionally, lipoxin epimers can be formed under the influence of aspirin treatment, as described before. Lipoxins exert their anti-inflammatory effects through signals generated by binding to a high-affinity, G protein-coupled lipoxin A4 receptor (ALX)/formyl peptide receptor, but they have also been shown to interact with other receptors such as the G protein-coupled receptor 32, the Aryl hydrocarbon receptor, the estrogen receptor, and the high affinity cysteinyl leukotriene receptor. A large body of experimental, preclinical, and clinical data have now established the anti-inflammatory and proresolution properties of lipoxin and related receptors. Studies in animal models show that inflammation-related conditions of renal and respiratory diseases could be brought under control through treatment with lipoxins. Low-dose acetylsalicylic acid (ASA) has been shown to provide anti-inflammatory effects by inducing the production of endogenous lipoxin. It is believed that drugs such as aspirin, which can trigger anti-inflammatory lipoxin synthesis, will have more broad applications in the future than the currently used anti-inflammatory drugs. The relationship between inflammation and cancer is well established and regular use of aspirin in cancer patients has shown promising results [9,10,11]. For this reason, ASA could have beneficial effects in the progression of NASH and, due to its antiplatelet activity, it could be particularly indicated in subjects with NAFLD, who often have an increased cardiovascular risk in the context of the metabolic syndrome.

Therefore, this study is aimed at evaluating, in an animal model, the effect of two different approaches, i.e., MitoQ and ASA, on NASH progression.

## 2. Materials and Methods

### 2.1. Animals and Treatment

In 2-month-old C57BL/6J mice, steatohepatitis was induced by 4-week administration of a diet poor in methionine (reduced by 66% compared to standard diet), deficient in choline, and fat-rich (46% kcal from fat), called MCDHFD [12,13]. Although mouse models of NASH, whether they are dietary, genetic, or toxin based, resemble many features of human NASH, none mimics the human state completely; for instance, while hepatic ballooning is a common occurrence in human NAFLD/NASH, it rarely occurs in mice [14]. MCDHFD induces NAFLD and NASH in male C57BL6/J mice in an accelerated manner compared with the Western diet and the choline-deficient models and does not cause significant weight loss like a classical methionine–choline-deficient diet [15].

Four experimental groups were studied (5 animals per group): a standard control group, a group of animals fed with MCDHFD diet, and two experimental groups fed with MCDHFD diet and treated with oral lysine acetylsalicylate (3.2 mg/100 g/day, equivalent to the 100 mg daily dose of ASA in 70 kg humans used for cardiovascular prevention) or MitoQ (1 mg/100 g/day) [16,17]. At the end of 4 weeks’ treatment, the animals were sacrificed with anesthetic overdose and liver samples were obtained.

The experiments were carried out in accordance with the legislation of Italian authorities (D.L. 27/01/1992 116), which complies with European Community guidelines (CEE Directive 86/609) for the care and use of experimental animals. The experimental protocol was approved by the Institutional Animal Care and Use Committee.

### 2.2. Histopathologic Evaluation

All liver biopsy samples were formalin-fixed, paraffin-embedded, and routinely stained with hematoxylin–eosin. Steatosis was assessed in a semiquantitative way and expressed as percentage of hepatocytes involved. The presence of necroinflammatory foci was scored as absent, mild (less than 1 focus per 20× field), moderate (2 foci per 20× field), or severe (>2 per 20× field). All liver biopsies were coded and scored by a single pathologist who was blinded to clinical and biological data.

### 2.3. Quantitative Real-Time Polymerase Chain Reaction

Total RNA from liver was extracted using RNeasy Mini Kit (Milan, Italy) according to the manufacturer’s instructions and quantified using a NanoDrop Spectrophotometer at 260 nm. Total RNA (up to 1 ug) was reverse transcribed using iScript cDNA synthesis kit (Bio-Rad, Hercules, CA, USA). The expression of transforming growth factor-beta1 (TGFβ-1), collagen type 1a1 (Col1a1), superoxide dismutase 1 (SOD-1), superoxide dismutase 2 (SOD-2), glutathione peroxidase 1 (GPx-1), catalase, tumor necrosis factor α (TNFα), interleukin-6 (IL-6), interleukin-1β (IL-1β), interleukin 10 (IL-10), Matrix Metallopeptidase 2 (MMP-2), Matrix Metallopeptidase 9 (MMP-9), tissue inhibitor of Metalloproteinase-1 (TIMP-1), and Serpinb3 was determined using SYBR Green Mastermix (Bio-Rad, Hercules, CA, USA) and performed in a CFX Real-Time PCR Detection Systems (Bio-Rad, Hercules, CA, USA) using the primer sequences shown in Table 1. Actin was used as a housekeeping gene. The relative expression was generated for each sample by calculating 2^−ΔΔCt^.

### 2.4. Western Blot Analysis

Total protein contents (80 ug) from each liver extract, assayed for protein content using the BCA protein assay kit (Pierce, Rockford, IL, USA), were prepared at 4 °C in lysis buffer (150 mM NaCl, 10 mM Tris-HCl (pH 7.4), 1 mM EDTA, 1 mM EGTA, 2% Triton X-100) in the presence of phosphatase and protease inhibitors (Thermo Fisher Scientific/Pierce, Rockford, IL, USA) and were loaded on 12% polyacrylamide gel. The blots were probed with rabbit polyclonal anti-GPx-1 (RayBiotech, Peachtree Corners, GA, USA), rabbit polyclonal anti-SOD-1 (Abcam, Cambridge, UK), rabbit polyclonal anti-IL-10 antibody (GeneTex, Alton Pkwy Irvine, CA, USA), and monoclonal antibody anti-COX-2 (Cayman Chemical,Ann Arbor, MI, USA). Mouse monoclonal anti-β actin (Sigma-Aldrich, St. Louis, MO, USA) was used as housekeeping control. Antimouse and antirabbit horseradish peroxide-conjugated antibodies (Amersham, Arlington Heights, IL, USA) were used as secondary antibodies. Antigenic detection was carried out using enhanced chemiluminescent substrate (Euroclone, Milano, Italy). Quantitative densitometric values of each protein were normalized to β-actin and displayed in histograms, using the Alliance Q9 Atom imaging system (Uvitec, Cambridge, UK). Regarding GPx-1, SOD-1, and IL-10, the membranes were first probed with anti-IL-10, then with anti-β-Actin, followed by anti SOD-1 and anti-GPX-1. Regarding COX-2, the membranes were probed first with anti-β-Actin and then with anti-COX-2. The experiments were performed in triplicate obtaining similar results.

### 2.5. Analysis of 15-Epi-Lipoxin A4 in Liver

Quantitative analysis of 15-epi-lipoxin A4 in the supernatant of liver homogenates was determined with an enzyme-linked immunosorbent assay from Neogen following the manufacturer’s instructions (Neogen Corp., Lexington, KY, USA). Briefly, 100 µL sample was diluted with 200 µL methanol and then diluted with 1.5 mL water. This was acidified to pH 3.5 with 1 N HCl. The sample was passed through a C18 Sep-Pak^®^ light column (Waters^®^ Corporation) preconditioned by washing with 2 mL methanol followed by 2 mL water. Following the sample, the column was washed with 5 mL water followed by 5 mL hexane. 15-epi-lipoxin A4 was eluted with 2 mL methyl formate and the methyl formate was evaporated with an N2 stream. The residue was reconstituted with 1 mL of diluted extraction buffer and assayed for 15-epi-lipoxin A4 content, following the manufacturer’s instructions of ELISA assay.

### 2.6. Statistical Analysis

Values are expressed as the mean ± SEM of the number of experiments (*n*). Statistical significance between the experimental groups was determined using one-way ANOVA followed by Bonferoni’s test. Values for *p* < 0.05 were considered statistically significant.

## 3. Results

### 3.1. Effects of ASA and MitoQ on Liver Steatosis and Necroinflammation through Histopathology

**Figure 1:**
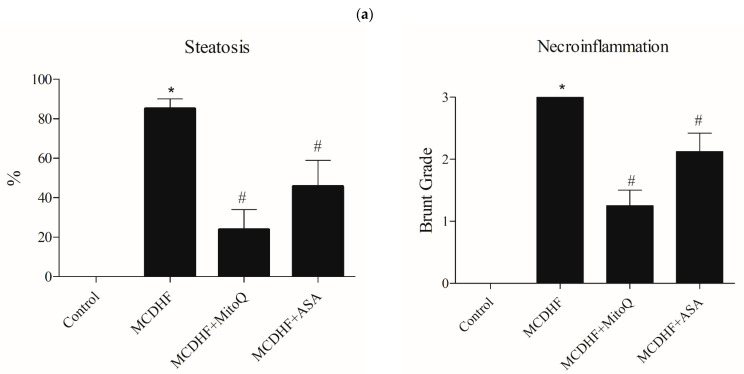

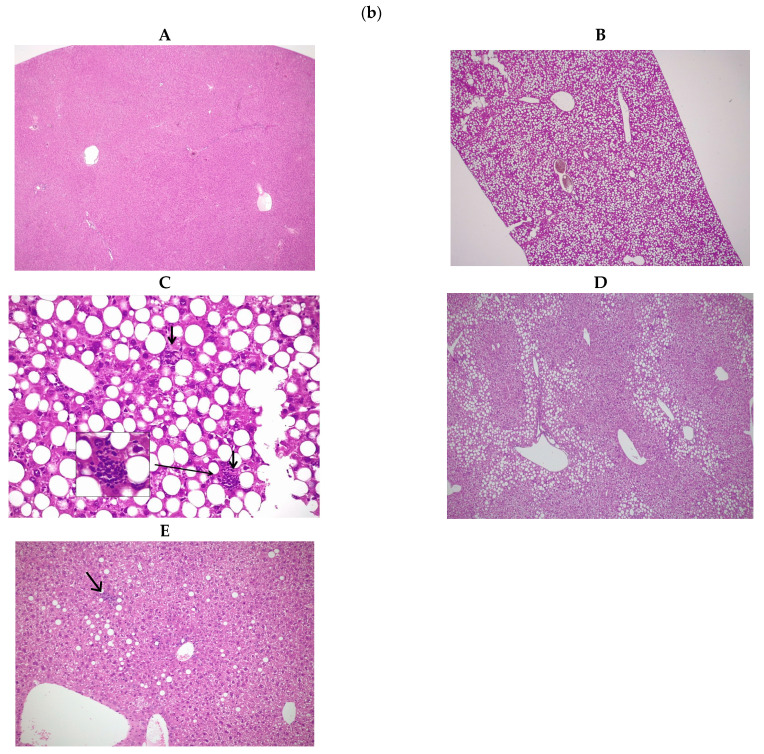
(**a**): Histological evaluation of steatosis and necroinflammation in livers from control, MCDHF, MCDHF + MitoQ, and MCDHF + ASA mice. * *p* < 0.05 vs. control; ^#^ *p* < 0.05 vs. MCDHF. In livers from MCDHFD mice, extensive steatosis and multiple inflammatory foci were observed; both steatosis and necroinflammation were improved by treatment with MitoQ and ASA. (**b**): (**A**): Control animal: neither steatosis nor inflammation are present; HE original magnification 2.5×; (**B**): MCDHF animal: severe macrovesicular steatosis involving more than 90% of hepatocytes; HE original magnification 2.5×. (**C**): MCDHF same animal as A: inflammatory foci are showed (arrows); HE original magnification 20× (40× in the insert). (**D**): MCDHF + MitoQ animal: less severe steatosis is evident, involving about 25% of hepatocytes, inflammation is absent; HE original magnification 5×; (**E**): MCDHF + ASA animal: steatosis is about 10% and inflammatory foci are rare (arrow); HE original magnification 20×.

The liver sections of control mice showed normal liver. The livers obtained from MCDHFD mice showed extensive steatosis and multiple inflammatory foci, which were significantly reduced by treatment with MitoQ and ASA (Figure 1a,b).

### 3.2. Effects of ASA and MitoQ on Liver Expression of Genes Associated with Inflammation, Oxidative Stress, and Fibrosis

**Figure 2:**
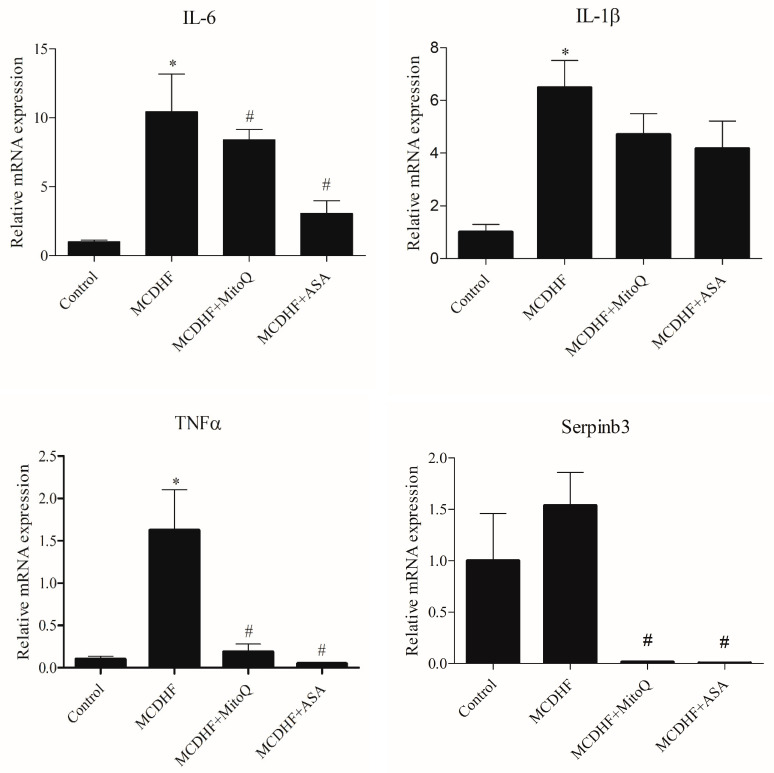

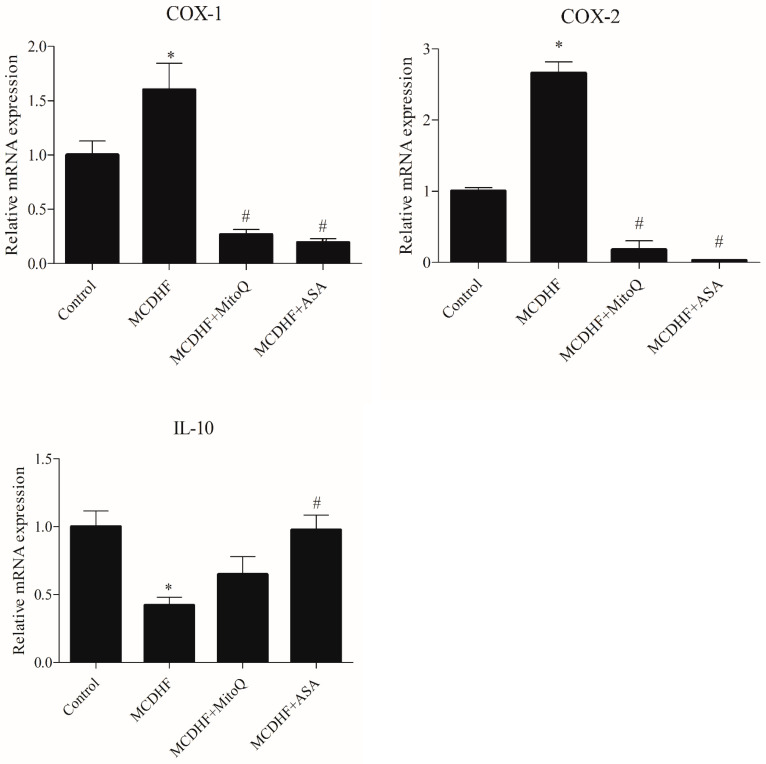
mRNA expression of IL-6, IL-1β, TNFα, Serpinb3, COX-1, COX-2, and IL-10 in livers from control, MCDHF, MCDHF + MitoQ, and MCDHF + ASA mice. * *p* < 0.05 vs. control; ^#^ *p* < 0.05 vs. MCDHF. The expression of proinflammatory cytokines TNFα, IL-1β, IL-6, Serpinb3, COX-1, and COX-2 was higher in MCDHFD mice compared to controls. Treatment with MitoQ and ASA determined a significant decrease in TNFα, IL-6, Serpinb3, COX-1, and COX-2 expression. Anti-inflammatory cytokine IL-10 expression was reduced in MCDHFD mice; treatment with ASA restored its levels in MCDHFD animals.

Higher gene expression of TNFα, IL-1β, IL-6, Serpinb3, COX-1 and COX-2, and proinflammatory cytokines was observed in MCDHFD mice compared to controls. MitoQ and ASA determined a significant reduction in hepatic inflammation, as suggested by a decreased gene expression of TNFα, IL-6, Serpinb3, COX-1, and COX-2. On the contrary, anti-inflammatory cytokine IL-10 gene expression was reduced in MCDHFD mice, but its levels were restored in mice treated with ASA (Figure 2).

**Figure 3:**
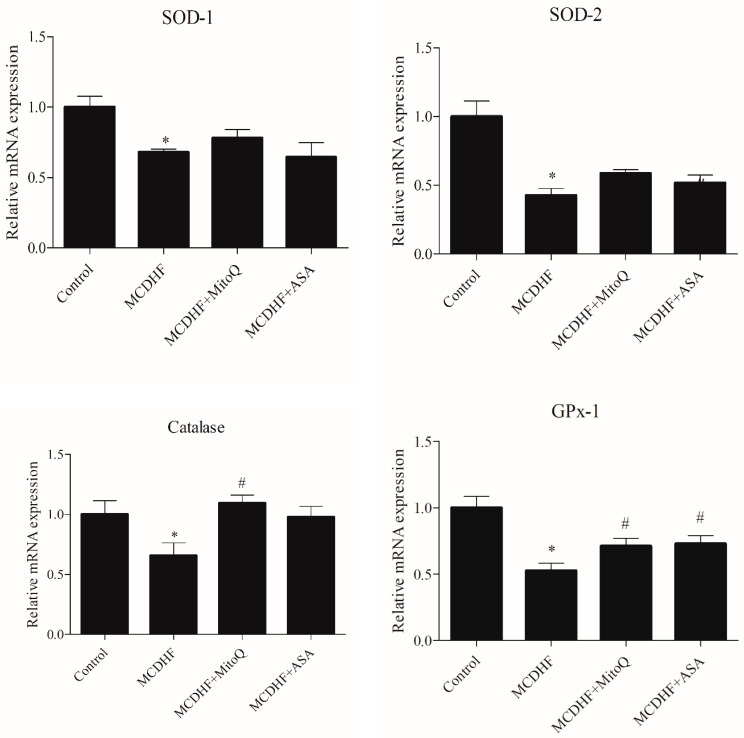
mRNA expression of SOD-1, SOD-2, catalase, and GPx-1 in livers from control, MCDHF, MCDHF + MitoQ, and MCDHF + ASA mice. * *p* < 0.05 vs. control; ^#^ *p* < 0.05 vs. MCDHF. In MCDHFD animals, the expression of antioxidant enzymes SOD-1, SOD-2, catalase, and GPx-1 was significantly reduced compared to controls; in MCDHFD mice, MitoQ significantly increased the expression of catalase and GPx-1, while ASA significantly increased the expression of GPx-1.

In MCDHFD mice, the expression of SOD-1, SOD-2, catalase, and GPx-1 enzymes defending the cell against oxidative stress was significantly impaired compared to control animals. In MCDHFD mice, treatment with MitoQ significantly increased the expression of catalase and GPx-1, and treatment with ASA significantly increased the expression of GPx-1 (Figure 3).

**Figure 4:**
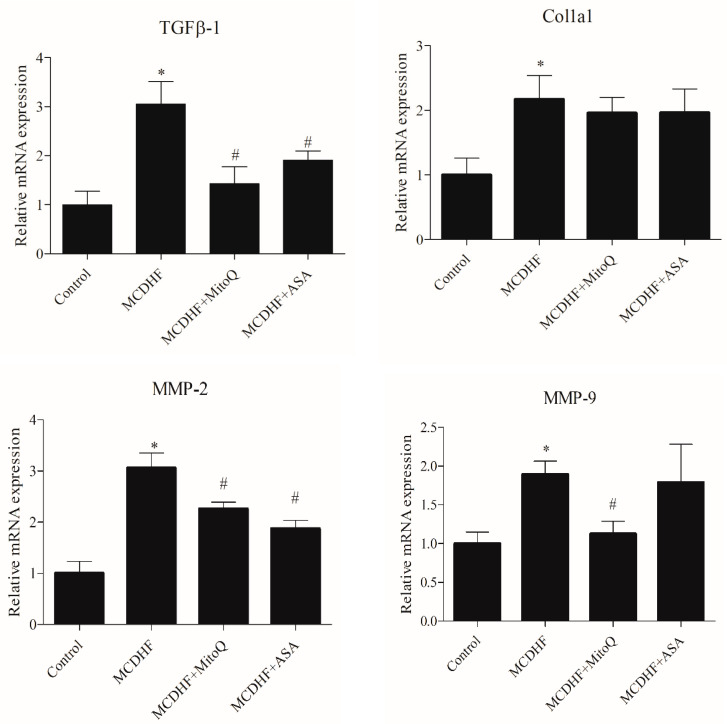

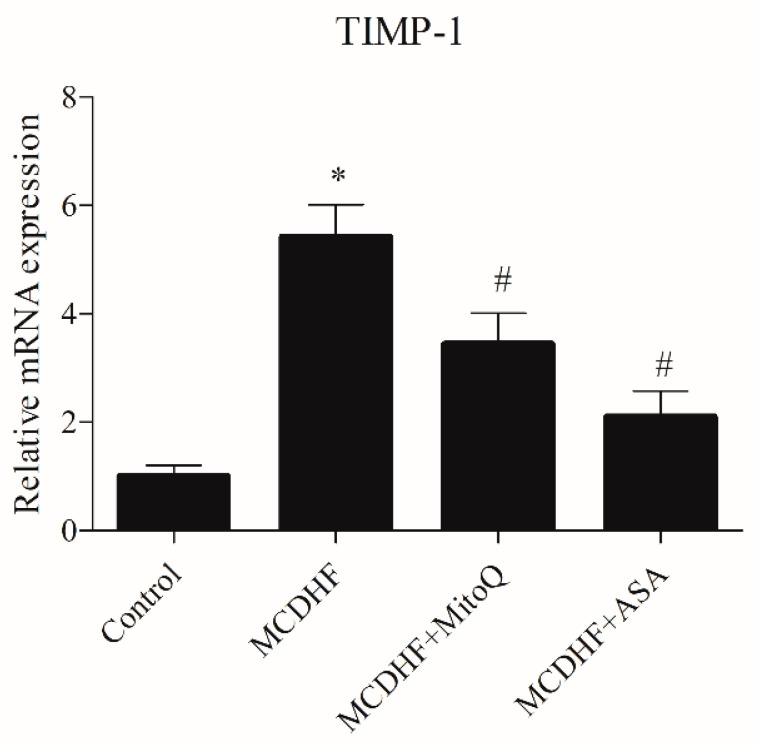
mRNA expression of Col1a1, TGFβ-1, MMP-2, MMP-9, and TIMP-1 in livers from control, MCDHF, MCDHF + MitoQ, and MCDHF + ASA mice. * *p* < 0.05 vs. control; ^#^ *p* < 0.05 vs. MCDHF. In MCDHFD mice, expression of genes associated with liver fibrosis, i.e., Col1a1, TGFβ-1, TIMP-1, MMP-2, and MMP-9, was significantly higher than in controls; treatment with MitoQ decreased the expression of TGFβ-1, TIMP-1, MMP-2, and MMP-9, while treatment with ASA decreased the expression of TGFβ-1, TIMP-1, and MMP-2.

Gene expression of Col1a1, TGFβ-1, TIMP-1, MMP-2, and MMP-9, which are associated with liver fibrosis, was significantly higher in MCDHFD mice than in control animals. In MCDHFD animals, MitoQ decreased the expression of TGFβ-1, TIMP-1, MMP-2, and MMP-9, while ASA decreased the expression of TGFβ-1, TIMP-1, and MMP-2 (Figure 4).

### 3.3. Effects of ASA and MitoQ on Protein Expression of SOD-1, GPx-1, IL-10, and COX-2 in Liver

**Figure 5:**
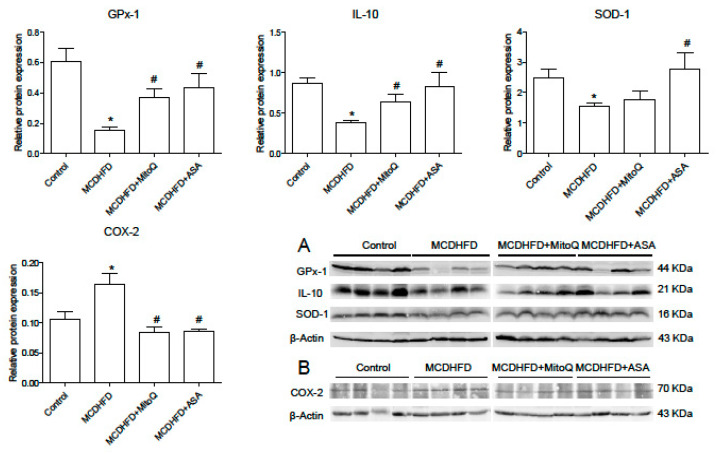
Protein expression of (A) GPx-1, SOD-1, IL-10, and (B) COX-2 in livers from control, MCDHF, MCDHF + MitoQ, and MCDHF + ASA mice. * *p* < 0.05 vs. control; ^#^ *p* < 0.05 vs. MCDHF. The experiments were performed in triplicate. In MCDHFD animals, GPx-1, SOD-1, and IL-10 hepatic expression was impaired compared to controls. Treatment with MitoQ and ASA both increased the expression of GPx-1 and IL-10, while only ASA increased the expression of SOD-1. In MCDHFD mice, both MitoQ and ASA normalized the expression of COX-2.

In liver from MCDHFD mice, GPx-1, SOD-1, and IL-10 expression was impaired compared to control animals. In MCDHFD mice, MitoQ increased the expression of GPx-1 (*p* < 0.05), while ASA increased the expression of both GPx-1 and SOD-1. In MCDHFD animals treated with both MitoQ and ASA, an increase in IL-10 protein expression was also observed. In MCDHFD mice, COX-2 expression was increased compared to controls, but it was reduced in animals treated with MitoQ or ASA (Figure 5).

### 3.4. Effects of ASA and MitoQ on Hepatic and Serum 15-epi-Lipoxin A4

**Figure 6:**
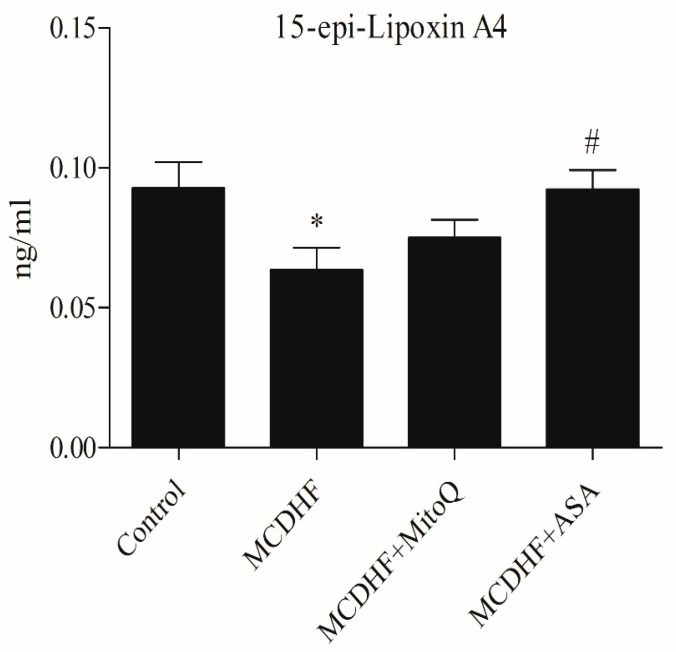
Liver 15-Epi-lipoxin A4 levels in control, MCDHF, MCDHF + MitoQ, and MCDHF + ASA mice. * *p* < 0.05 vs. control; ^#^ *p* < 0.05 vs. MCDHF. In MCDHFD animals, hepatic levels of anti-inflammatory 15-epi-Lipoxin A4 were reduced; ASA, but not MitoQ, normalized the levels of 15-epi-Lipoxin A4 in liver.

In liver from MCDHFD mice, compared to controls, anti-inflammatory 15-epi-Lipoxin A4 levels were reduced. Treatment with ASA, but not with MitoQ, restored the levels of 15-epi-Lipoxin A4 in livers from MCDHFD mice (Figure 6).

## 4. Discussion

NASH is a chronic liver disease, characterized by diffuse fatty infiltration with concomitant inflammation and hepatocyte injury, that can evolve into liver cirrhosis. Moreover, because of its high prevalence in the general population, NAFLD may coexist with other liver diseases, increasing the severity of fibrosis, risk of cirrhosis, and hepatocellular carcinoma in patients with chronic hepatitis B, chronic hepatitis C, and alcohol-related liver disease. Lifestyle interventions, including dietary calorie restriction and exercise, constitute the central pillar of NAFLD management, but only a small part of patients is able to sustain a weight loss and a change in lifestyle over meaningful periods. On the other hand, even if, among the various drugs used in trials for the treatment of patients with NASH, some such as vitamin E, pioglitazone, and saroglitazar show promising results, to date, no therapy has been officially approved for NAFLD and NASH [18]. Drugs that intervene in the regulation of inflammation and oxidative stress could provide an effective therapeutic strategy for NASH. Therefore, we investigated the efficacy of ASA and MitoQ against NASH and the possible mechanisms involved by using a mouse model of NAFLD. Low-dose ASA, by inducing lipoxin production, could reduce inflammation in NASH [10]. MitoQ, in view of the predominant role of mitochondria in the production of ROS, could also have anti-inflammatory and antifibrotic effects.

Our study demonstrates that treatments with ASA and MitoQ reduce steatosis and inflammation in MCDHFD mice. It is difficult to determine whether mitochondrial dysfunction and oxidative stress are primary events or a consequence of inflammation in NASH development; however, as suggested by the increased gene expression of antioxidant catalase and GPx-1 and protein expression of GPx-1 and SOD-1, the beneficial effects of ASA and MitoQ seem to be correlated with a decrease in oxidative stress [7]. MitoQ is as mitochondria-targeted antioxidant, which acts to prevent mitochondrial dysfunction; therefore, the anti-inflammatory effects of MitoQ in NASH could be mainly a consequence of its anti-oxidant activity. Similarly, the beneficial antioxidant properties of MitoQ have been demonstrated in rats with alcoholic steatohepatitis [19]. On the other hand, we can speculate that ASA primarily suppressed the inflammatory processes and this was associated with a normalization of the levels of antioxidants in MCDHFD mice liver. In liver of MCDHF mice, ASA restored the levels of 15-epi-Lipoxin A4, which could be the main anti-inflammatory pathway involved. On the other hand, the beneficial effects of ASA on inflammation can also be explained by the decreased expression of COX-1, COX-2, TNFα, and IL-6 and the increased expression of anti-inflammatory IL-10. The inhibitory effect of ASA on COX-2 expression has also been shown in other recent studies, but the decrease in COX-2 expression could be promoted by the reduced levels of TNF-α as well [20,21,22,23,24]. In line with the results of our study, it has been shown that, in mice fed with a methionine- and choline-deficient diet, hepatic expression of COX-2 messenger RNA and protein was significantly higher than controls, paralleling the increase in the levels of TNF-α and IL-6 and the development of steatohepatitis; pharmacological inhibition of COX-2 ameliorated the severity of experimental steatohepatitis [25]. Nevertheless, it should be underlined that, regarding the impact of COX-2 on the development of NASH, data are controversial, as other studies suggested that PGE2 suppresses fibrogenesis, and COX-2 overexpression in hepatocytes may protect against NASH development by attenuating steatosis and inflammation [26,27].

Another study showed that in rats fed with a choline-deficient, L-amino acid-defined diet, ASA significantly attenuated liver steatosis, inflammation, and fibrosis, but the doses administered were higher than those commonly used by patients in chronic treatment with ASA, so data on the effectiveness of a low dose of ASA on NASH were lacking [28]. This is particularly important considering that the major risk factors for NASH include metabolic syndrome, abdominal obesity, insulin resistance, glucose intolerance or type 2 diabetes mellitus, and dyslipidemia, conditions often associated with cardiovascular complications that could require treatment with ASA as an antiplatelet drug at doses equivalent to the ones used in our animal model.

Moreover, in this study, we observed that Serpinb3 was strongly reduced as a result of treatments with ASA and MitoQ. The results are in line with the role of this molecule, which increases in oxidative stress conditions and can contribute to the fibrogenic progression of chronic liver disease [29,30]. In addition, experimental results in vitro have recently supported a driving role of SerpinB3 in the upregulation of the COX-2 positive loop, further supporting the study results [31].

We evaluated if the development of steatosis and hepatic inflammation was associated with an increased expression of profibrotic genes in liver. In MCDHFD mice, the expression of CollA1 and TGFβ-1 was increased. Treatment with both ASA and MitoQ significantly reduced TGFβ-1 expression. We have previously demonstrated that in cirrhotic rats MitoQ decreases the expression of Col1A1 and TGFβ-1 and improves liver fibrosis [16]. Since an extensive remodeling of the extracellular matrix occurs during hepatic fibrogenesis and TIMP-1 regulates hepatic extracellular matrix remodeling by MMPs, we evaluated TIMP-1, MMP-2, and MMP-9 gene expression. In liver from MCDHFD mice, an increased TIMP-1 and MMP-2 gene expression was downregulated by ASA and MitoQ treatment; ASA also significantly reduced MMP-9 expression. Although the role of MMPs is complex as the same MMP can have opposing effects based upon the cell type or tissue, consistent with these data, we have shown that in cirrhotic rats the improvement of fibrosis due to MitoQ administration was associated with a decrease in TIMP-1 and MMPs liver expression [16].

## 5. Conclusions

In conclusion, our data suggest that ASA and MitoQ reduce the progression of liver steatosis, inflammation, and fibrosis in an animal model of NASH. This, at least in part, seems to be secondary to a reduction in oxidative stress, as suggested by an increased expression of antioxidant enzymes. Due to these properties, ASA and MitoQ may be a useful supplement for the treatment of NASH.

## Figures and Tables

**Table 1:** List of primers used in mice liver tissues.

TGFβ1	Sense 5′-TTGCTTCAGCTCCACAGAGA-3′ Rev 5′-TGGTTGTAGAGGGCAAGGAC-3′
Col1a1	Sense 5′-AAA TCT GCA CAC TGC CAT GA-3′ Rv 5′-GCA TGT TCG AAA TCC AGT GA-3′
SOD-1	Sense 5′-AACCATCCACTTCGAGCAGA-3′ Rev 5′-TACTGATGGACGTGGAACCC-3′
SOD-2	Sense 5′-GCCTGCTCTAATCAGGACCC-3′ Rev 5′-GTAGTAAGCGTGCTCCCACA-3′
Catalase	Sense 5′-CACTGACGAGATGGCACACT-3′ Rev 5′-TGTGGAGAATCGAACGGCAA-3′
TNFα	Sense 5′-AGCCCCCAGTCTGTATCCTT-3′ Rev 5′-CTCCCTTTGCAG AACTCAGG-3′
GPx-1	Sense 5′-AGTCCACCGTGTATGCCTTC-3′ Rev 5′-CCTCAGAGAGACGCGACATT-3′
IL-6	Sense 5′-AGTTGCCTTCTTGGGACTGA-3′ Rev 5′-CAGAATTGCCATTGCACAAC-3′
IL-1β	Sense 5′-GAAATGCCACCTTTTGACAGTGAT-3′ Rev 5′-TTGGAAGCAGCCCTTCATCTT-3′
Serpinb3	Sense 5′-TCCTAGTGGGAGCCTAAGCA-3′ Rev 5′-ATCCCCCAGAAAGCTGAAGT-3′
COX-1	Sense 5′-TCTGCCTCAACACCAAGACC-3′ Rev 5′-AGACAGACCCGTCATCTCCA-3′
COX-2	Sense 5′-TATGCCACCATCTGGCTTCG-3′ Rev 5′-GTTGCTCATCACCCCACTCA-3′
IL-10	Sense 5′-CCAAGCCTTATCGGAAATGA-3′ Rev 5′-TTTTCACAGGGGAGAAATCG-3′
MMP-2	Sense 5′-CCA ACT ACA ACT TCT TCC CCC-3′ Rev 5′-CGA GCA AAA GCA TCA TCC AC-3′
MMP-9	Sense 5′-CCCTGGAACTCACACGACAT-3′ Rev 5′-TGGTTCACCTCATGGTCCAC-3′
TIMP-1	Sense 5′-ATGCCCACAAGTCCCAGAAC-3′ Rev 5′-TACGCCAGGGAACCAAGAAG-3′

## Data Availability

The authors confirm that the data supporting the findings of this study are available within the article.
